# Data in Diabetic Foot Care: From Current State to a Management Framework for Implementation

**DOI:** 10.3390/jcm14248674

**Published:** 2025-12-07

**Authors:** Iztok Štotl

**Affiliations:** 1 Department of Endocrinology, Diabetes and Metabolic Diseases, University Medical Centre Ljubljana, 1000 Ljubljana, Slovenia; iztok.stotl@guest.arnes.si; 2 Faculty of Medicine, University of Ljubljana, 1000 Ljubljana, Slovenia

**Keywords:** diabetes, diabetic foot, electronic health record, governance, prediction models, digital practice guidelines, guidelines definition language, diabetes registries, fair, quality of care

## Abstract

**Background/Objectives**: The healthcare data sector is experiencing unprecedented growth, fueled by advances in genomics, medical imaging, and wearable devices. The convergence of universal data standards now provides the common ground needed to translate this data into medical advances. However, a significant implementation gap persists, preventing effective deployment in routine clinical practice, particularly in specialized areas like diabetic foot care. **Methods**: This paper examines the opportunities presented by modern data methodologies to bridge this gap, contextualized within diabetic foot care, where the paramount goals are patient well-being, tissue preservation, and amputation prevention. **Results**: The analysis indicates that the synergy of interoperable data and advanced management tools is poised to fundamentally transform healthcare delivery. Interdisciplinary collaboration is identified as the foundational element enabling the timely, coordinated, and evidence-based interventions necessary to achieve critical clinical objectives. **Conclusions**: The pivotal challenge has shifted from technological capability to effective implementation. Leveraging modern data methodologies is essential for translating potential into tangible improvements in diabetic foot outcomes. In this context, collaborative data management must be recognized as a critical treatment modality itself. Here, “data is tissue”; it must be managed with the same urgency and care to enable success.

## 1. Introduction

Data are undoubtedly profoundly shaping our society and daily lives while becoming one of the world’s most valuable resources [1]. It is fundamentally transforming how we approach and solve contemporary problems to such an extent that the question is often no longer whether to use it, but how to use it effectively. This paradigm is directly applicable to the diabetic foot, which continues to adversely affect the lives of patients with diabetes [2]. The quest for data utilization in foot care started in earnest with the landmark St. Vincent Declaration of 1989 [3]. While its primary aim was to improve clinical outcomes, its ambitious call to reduce diabetes-related amputations by half created an undeniable imperative for systematic data collection. This mandate established the foundational pathway for modern data goals, transforming the management of diabetic foot complications from a realm of anecdotal experience into one that increasingly relies on robust data to track progress, identify at-risk populations, and ultimately achieve its visionary target. Despite its importance and proliferation of scientific publications, data-driven approaches to diabetic foot care remain markedly underutilized in routine practice, creating a critical gap in management. This review highlights some of the opportunities presented by modern data methodologies and reviews their current developments in the field of diabetic foot.

## 2. Unlocking the Potential of Electronic Health Records (EHR)

Arguably, the most critical element for using clinical data successfully is at the very moment of its creation—by capturing it effectively when patients interact with the healthcare system. Paradoxically, although EHRs form the central nervous system of modern healthcare, a core component of diabetic foot information often remains paralyzed. Invaluable data is either trapped in unstructured clinical notes or sequestered within proprietary, non-standard data vaults. This renders it less reusable, stifling collaboration by limiting our ability to communicate, generate insights, and build advanced tools. Moreover, the system design philosophy of current EHR implementations often prioritizes bureaucratic and billing requirements over clinical utility, creating systems that physicians must work around rather than with [4]. This misalignment does not just hamper data creation but has also contributed significantly to clinician burnout [5,6,7]. The path forward requires a fundamental reimagining of EHRs as clinical tools first and administrative instruments second [8].

Moving toward truly effective EHR systems requires addressing several fundamental requirements. First and foremost is usability—systems must be designed to support rather than disrupt clinical reasoning. This means intuitive interfaces that mirror clinical thought processes, documentation tools that capture necessary information without redundancy, and decision support that appears at the right time in the workflow. Equally important is the need for systems that can evolve with medical knowledge. Current EHR architectures often make implementing new guidelines or quality measures a years-long technical project. Future systems need to be more agile, allowing clinical leaders to update protocols and decision rules without extensive information technology (IT) support. Perhaps most crucially, we need to recognize that better EHR design is not just a technical challenge but a cultural one. It requires breaking down silos between clinicians and technologists, valuing workflow efficiency as highly as regulatory compliance, and creating continuous feedback loops between system users and designers.

### 2.1. Establishing a Common Ground for Health Data

Interoperability is the ability of IT systems to exchange and make use of diverse data. A vital advancement in health informatics is the field’s recent consolidation around principal open data standards [9,10]. Common ground provided by data standards supports user-oriented digital services that are open for local and central innovations based on distributed governance [11]. These standards are open, supported by active communities, and have been proven effective through extensive implementation in their respective domains:

- **Terminologies** (Systematized Nomenclature of Medicine—Clinical Terms (SNOMED CT) [12], Logical Observation Identifiers Names and Codes (LOINC) [13], Orphanet Rare Disease Ontology (ORPHA) [14], etc.) act as the “universal medical dictionary”. They give every medical concept—like “Charcot foot” or a “neuropathy test”—a unique code. This ensures that when different systems use the word “Charcot foot,” they are all referring to the exact same condition.
- **OpenEHR** [15] is like the “architect’s detailed blueprint”. It focuses on how to design the optimal, future-proof digital patient record itself. It allows clinicians to define precisely what information should be captured and stored in a way that computers can understand unambiguously.
- **Fast Healthcare Interoperability Resources (FHIR)** [16] (pronounced “fire”) is designed for the “secure delivery service”. Once the data is stored (using the blueprint), FHIR provides a modern, standard way to quickly and securely package and send that information between different systems, like from a hospital’s computer to a patient’s phone app.
- **The Observational Medical Outcomes Partnership (OMOP) Common Data Model (CDM)** [17] is the “standardized research frame”. Its job is to help us learn from diverse types of health information. It takes data from all sorts of different systems and translates it into a common format, allowing researchers to run large-scale analyses to find better treatments and understand diseases. Additionally, the OHDSI community provides associated tools for preparing data and conducting research.

**Figure 1:**
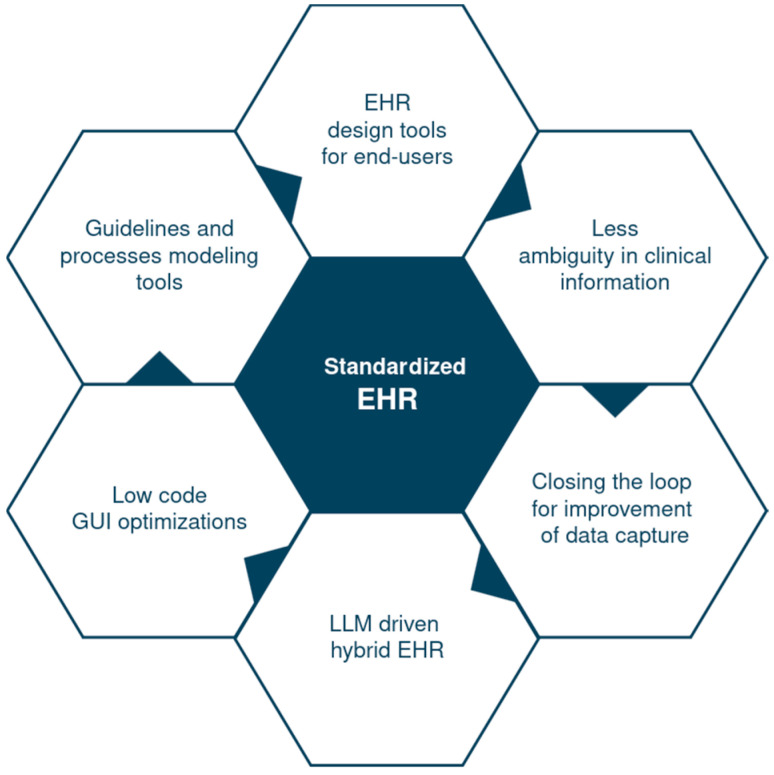
Standardized EHR—the common ground of many functionalities. EHR—electronic health record, GUI—graphical user interface; LLM—large language models.

The adoption of common EHR data standards establishes a foundational framework that reduces ambiguity in clinical information across different healthcare providers. It enables interoperability, ensuring that the data are structured and defined consistently, enabling seamless communication and many additional functionalities (Figure 1). One of the key benefits of this standardization is the reusability of analytical solutions. Tools for data analysis, reporting, and population health management can be applied seamlessly across data from different EHR systems without requiring costly and time-consuming adaptations, as they operate on a uniform data structure [18]. Furthermore, standardized data empowers end-users in collaboration on EHR design. Clinical guidelines, process models, and user-friendly design tools can be shared and reused effectively because they are built upon the same underlying framework [19]. This common background also facilitates low-code/no-code graphical user interface optimization, allowing clinicians and administrators to customize their own workflows and interfaces without deep programming expertise [20].

The potential of this standardized environment can be significantly amplified by integrating an LLM (Large Language Model)-driven hybrid approach to EHRs [21], which refers to systems that combine structured and unstructured data. Hybrid models extend established data standards with the flexibility offered by LLMs and emerging small language models [22]. After summarizing the rich information buried in the vast amount of information contained in free-text clinical notes, progress reports, and physician narratives, they can provide highly personalized and relevant clinical decision support, flagging potential medication interactions or recommending evidence-based guidelines specific to that patient’s unique narrative. An LLM-driven hybrid approach to EHR does not replace the need for common data standards [23]; it builds upon them. The data standards provide clean, reliable, structured data, while the LLM acts as an intelligent layer that can harness the power of unstructured text, creating a more complete, intuitive, and powerful clinical environment.

### 2.2. Technological Ability Is Not Enough

The recent randomized trial comparing digital Clinical Decision Support System versus traditional foot examinations found equivalent patient satisfaction but superior clinical utility, with 100% versus 2% risk classification completeness [24]. Healthcare professionals reported that the digital tool provided guideline-based, structured examinations and thorough documentation, though better EHR integration was needed. Optimizing EHR systems should not be seen only as a technical upgrade; it is a pivotal moment that reshapes the entire ecosystem of care. At its heart, this transformation is about standardizing clinical practice itself, which demands a system-wide commitment to new forms of governance and collaboration [25].

The existence of mature IT infrastructure and emerging standards, such as the European Electronic Health Record Exchange Format (EEHRxF) built upon the legal foundation of the European Health Data Space (EHDS) [26], provides the technical capacity for data exchange. However, significant implementation and interoperability challenges remain [27]. Our end-user tools are often unfit for clinical practice [28,29], leading to insufficient data quality and incomplete data. In a recent 2025 survey of clinicians on the future of clinical practice, only around a third thought their institutions were performing well in providing digital tools, including artificial intelligence (AI) support [30]. A study of an EHR optimization program in two major Danish hospitals revealed that 69% of physicians disagreed or strongly disagreed that the system facilitated their work, while only 15% agreed or strongly agreed [31,32]. This challenge is also encountered in other countries [33]. Large-scale EHR implementations are inherently defined by 21 sociotechnical tensions revolving around people, power, resources, system, and vision [34]. Acknowledging these predictable tensions is the first step to mitigating their impact across the entire transition. There is limited knowledge on how to effectively organize and manage such optimization initiatives. For implementation to succeed, it is critical to balance standardization with local flexibility and to ensure clinical users are not merely involved but wield genuine influence throughout the process [35,36]. Ultimately, success must be evaluated through rigorous usability assessments that employ validated and reliable measures [37,38].

### 2.3. Bridging the Clinician-IT Designer Divide

Unclear frameworks, theories, and guidelines for healthcare professionals (HCPs) inclusion in EHR design lead to suboptimal involvement, despite its importance [39]. Currently, EHR design tools are predominantly used by IT experts, and there is a lack of international or cross-vendor collaboration among healthcare providers. Moreover, the entry points for healthcare providers to engage in EHR design collaboration remain unclear. Healthcare workers generally lack familiarity with the digital concepts necessary to implement needed changes. They need tools to think with—tools that aim to provide a common language, enabling them to debate what works, when, and why [40]. The integration of eHealth competencies into continuing professional development frameworks remains a significant and largely unaddressed gap [41,42]. Data management and AI literacy are now fundamental skills for healthcare practitioners. They must transition from passive consumers to proactive leaders—actively engaging with the technology, understanding its principles, and championing its ethical and equitable implementation to advance patient care [43]. Consequences of implementing the EHDS framework on doctors, patients, and the provision of healthcare are not clear [44,45]. To tackle this, the European Xpanding Innovative Alliance project (XiA) aims to develop and disseminate high-quality training to empower healthcare professionals, IT providers, and developers with the practical skills needed for eHealth readiness. Its core goal is to foster a culture of interoperability and ensure the workforce is prepared to successfully embrace and implement EHDS-related standards [46].

Overcoming technical barriers demands a dual strategy: First, applying a human factors approach to simplify interfaces, ease information retrieval, and minimize complex tasks [47]. Second, proactively deploying AI to reduce cognitive load and administrative work. To ensure this AI is effective and safe, vendors and users must collaborate closely on its development and implementation [48].

## 3. Collaborative Care in the Digital Age

**Figure 2:**
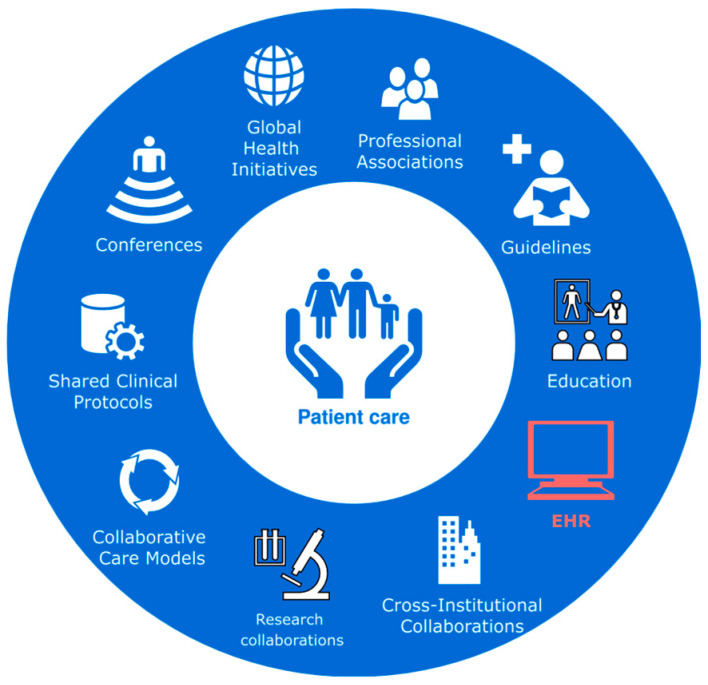
EHR—one of the cornerstones for healthcare practitioners’ collaboration.

Collaboration at local, national, and international levels is a powerful tool for improving patient care. EHR systems play a critical role in this process and should foster trust, transparency, and collaboration between patients and healthcare professionals (HCP) (Figure 2). These systems must be designed to be user-friendly, supporting clinicians in their tasks and reducing the burden of administrative work [8]. To effectively reuse EHR data, structured data capture is essential. By facilitating structured recording, electronic care pathways can reduce documentation burden and eliminate the need for manual data extraction for quality registries. For success, these systems must be closely aligned with clinical workflows [49].

The standardization of EHR content is a powerful mechanism for standardizing clinical practice itself—a change that can yield dramatic and far-reaching consequences [25]. The practice of medicine has always been inherently collaborative, but EHR systems have the potential to elevate this collaboration to unprecedented levels. In contemporary healthcare settings, these digital platforms serve as the connective tissue linking diverse care teams across specialties and institutions. They facilitate not just information sharing but also the potential implementation of evidence-based medicine at the point of care.

Consider how professional medical associations develop and disseminate clinical guidelines [50]. These painstakingly crafted documents represent the distillation of global medical knowledge, yet their impact in IT systems is often blunted by implementation challenges. When housed within intuitive EHR systems, these guidelines could transform from static PDF documents into dynamic clinical decision support tools. This transition would enable automatic risk stratification, context-aware recommendations, and real-time quality metric tracking—all seamlessly integrated into the clinician’s natural workflow.

### A Conceptual Case Study: Reimagining Diabetic Foot Care Through Digital Integration

With the involvement of over 100 experts from more than 60 countries, the International Working Group on the Diabetic Foot (IWGDF) guidelines are a great example of the benefits of international collaboration in improving patient care [51]. These guidelines serve 2.9 billion people globally, with translations currently available in over 25 major languages. Although digital transformation undoubtedly offers numerous opportunities, there are many open questions regarding how clinical guidelines could be translated into a ‘digital language’ based on the common data ground that is starting to emerge.

How or where should a clinical society debate digital aspects of its work? In the current circumstances, where clinical knowledge governance models are still in development and do not provide a clear answer as to who and where should digitize international guidelines and clinical content, the professional organizations like the IWGDF seem more than suitable to take on the role of a hub for organizing and establishing such a digital consensus on an international level. Yet these comprehensive guidelines remain trapped in formats that limit their clinical utility. When a podiatrist evaluates a diabetic patient, they must mentally cross-reference multiple PDF documents while documenting in an EHR system that may not align with guideline recommendations. This cognitive burden could be dramatically reduced if the guidelines were digitally encoded directly into the EHR as structured clinical pathways.

The technical standards for digitizing guidelines, such as the Guideline Definition Language [52] and Clinical Practice Guidelines on FHIR [53], are already mature and enable the transformation of narrative text into actionable, machine-readable formats. However, the organizational hurdles to widespread implementation are non-trivial, necessitating standardized translation methods, intuitive tools for clinicians, and flexible implementation frameworks that accommodate local variations.

How can the professional societies ensure the wide deployment of their decisions? The current form of guidelines [50] (PDF and websites) is not machine-readable and lacks a direct interface for integration into existing information systems, leading to slower and less effective adoption of best practices in the software solutions used in everyday practice. Standardized EHR definitions and computable clinical practice guidelines (CPGs) have the potential to significantly enhance the adoption of guidelines by healthcare providers. If created in the right form, they can be quickly and timely integrated into existing information systems worldwide with less additional effort for implementation. Such standardization would also unify the form and entry of primary data, improving its use for different secondary purposes, such as registries and analytics.

To address and bridge the significant knowledge gap between clinical experts and IT specialists, effective communication must be enabled, allowing both groups of experts to contribute insights, provide feedback, and approve final decisions. Collaborative efforts by a multidisciplinary group of experts ensure that more relevant questions are addressed and answered comprehensively. To achieve the most effective knowledge exchange and validation between these diverse expert groups, a multi-layered knowledge representation framework should be implemented.

The suitable framework proposed by Boxwala et al. [54] is depicted in Table 1 and introduces four successive layers that progressively structure knowledge to facilitate structured, hierarchical, and accessible communication, enabling seamless collaboration and alignment across disciplines. The native guidelines are initially provided as a narrative description (L1 knowledge level). These are refined into a semi-structured, human-readable format (L2 knowledge level), authored primarily by domain experts. This format is specifically designed to facilitate effective communication between clinical domain experts and knowledge engineers, ensuring clarity and alignment across both groups. This version is then further transformed by a knowledge engineer with expertise in clinical decision support into a structured, computer-readable format (L3 knowledge level). It should be specified with sufficient structure so as to make it commutable and precise, with the objective of communicating the knowledge in the guidelines from knowledge engineers to local clinical decision support system implementers. Such structured knowledge finally enables local implementations in an executable form (L4 knowledge level). The L2 and L3 levels serve as the final digital guideline deliverables, acting as a bridge between the textual guidelines (L1) and the practical implementation of local informatics solutions (L4).

The development of information systems in healthcare often involves a struggle to consolidate experts’ disparate knowledge trajectories into a synergistic whole [55]. A significant opportunity for improvement in this process lies in incorporating direct participation from international professional societies. The integration of rapidly translated clinical guidelines with advanced analytical insights would establish the necessary foundation for learning healthcare systems [56]. Despite its promise, this concept remains underimplemented on a global scale. While global clinical recommendations about diabetic foot increasingly incorporate hard evidence about mobile applications and electronic device use into their recommendations [51,57], they largely remain silent on substantial advancements in complex machine learning predictive models and other eHealth innovations. This represents a clear, untapped potential and a missed opportunity to significantly bolster local efforts with expert-backed tools and recommendations.

Modern, complementary approaches like adaptive mirroring balance central and local national needs in IT infrastructure [11]. They would also benefit from modular and reusable knowledge components, furnishing local and national consolidation processes with dynamic reconfiguration based on input from international professional societies.

## 4. Prediction Models and AI

Machine learning is a dominant and crucial subset of AI that is used to build prediction models as one of its practical applications—to forecast a future outcome or assign a probability of events. A recent review highlighted the significant potential of diabetic foot risk prediction models and the expansion, diversification, and in-depth development of research dedicated to them [58]. By integrating diverse predictive factors, including medical history, foot exams, and lab results, the models provide a comprehensive assessment of diabetic foot risk. They are effective tools for accurately identifying risk factors and guiding early interventions, with the potential to reduce the incidence of diabetic foot ulcers. This approach is a prime example of how patient-specific data enables a shift from a one-size-fits-all model toward precisely tailored interventions [59]. Model-created probability estimates can optimize clinical workflows, leading to substantial resource savings. A sustainability pilot study demonstrated how this approach could potentially halve the number of required foot screenings while maintaining patient safety, overcoming the inefficiencies of the current resource-constrained system [60].

Current machine learning applications in diabetic foot care primarily focus on thermal imaging and Internet of Things innovations [61]. Furthermore, AI-driven predictive analytics—powered by wearable technologies such as continuous glucose monitors, smart insoles, and temperature sensors—can identify early signs of diabetic foot ulcers, enable real-time monitoring, and generate early warnings [62]. By also integrating data on genetics, social and structural determinants [63], environment, and lifestyle, future models could achieve more accurate and personalized predictions [58].

With the availability of diverse data, the digital twin concept presents a promising opportunity through the creation of a virtual model of a patient’s condition, such as a wound. Such a model can simulate the healing process to predict outcomes and guide treatment [64,65,66]. By comparing the actual wound to its digital twin, caregivers can proactively identify non-healing wounds and make timely, personalized adjustments to therapy. This advanced technology could be further enhanced by coupling it with real-time data from diabetes digital health applications, which have already demonstrated the potential to improve disease outcomes in real-world settings [67]. Additionally, associating clinical outcomes with deeply phenotyped large-scale data will allow us to pursue a new generation of questions about disease [68].

The future development of these models will be driven by the continuous collection and analysis of new clinical data. However, scientific validity and technical support in the clinical setting are still lacking and need to be consolidated through high-quality randomized controlled trials specifically targeting data-driven interventions. Despite promising advances in the theoretical field and an explosive increase in annual publications about AI in healthcare worldwide [69,70], clinical translation persists as the primary challenge, preventing these clear achievements from reaching patients. Only around a third of clinicians think their institutions perform well in providing digital tools, including AI. They also consider institutional performance lower for AI training (30%) and AI governance (29%) [30]. Key barriers include failing to establish a common data ground (discussed in 2.1), overcoming regulatory compliance and standardization issues across healthcare systems [71], addressing governance issues [72], and achieving clinical workflow integration. Additionally, model explainability, prospective validation, and equitable implementation remain significant burdens [73,74,75].

## 5. Systemic Approach to Better Insights and Quality Improvement

As comprehensive informatics networks for diabetes care and research, national registries in Sweden [76] and Scotland [77] have been used effectively as clinical tools for risk assessment, monitoring, and comparison, thereby promoting improvement through measurement and encouraging clinical research with a focus on patient benefit. Despite the existence of best practices, considerable variation in the maturity and implementation of diabetes registries and data sources hinders the comparability of care quality and patient outcomes [78].

On the global level, international organizations like the World Health Organization (WHO), International Diabetes Federation (IDF), Organization for Economic Co-operation and Development (OECD), European Best Information Through Regional Outcomes in Diabetes (EUBIROD) and International Consortium for Health Outcomes Measurement (ICHOM) play a crucial role in developing global diabetes care measures and standards—such as the WHO’s HbA1c < 8% target—and facilitate cross-country comparisons through initiatives like the IDF Diabetes Atlas [79], OECD’s Health at a Glance [80], ICHOM outcome sets [81], and EUBIROD’s European data integration efforts [78]. However, significant challenges remain in data availability, quality, and implementation, particularly in low- and middle-income countries, where reporting is often limited to basic metrics like prevalence and mortality, lacking the detailed care quality and outcome indicators routinely available in high-income countries. A critical insufficiency of current efforts is that neither the OECD nor the IDF currently reports even on such basic parameters as HbA1c, which is the evidence-based, clinically recommended gold standard for evaluating diabetes care quality—thereby limiting meaningful international comparison and improvement of diabetes outcomes [82]. According to the 2025 review [83], a significant gap exists in the availability of specific PROMs (Patient-Reported Outcome Measures) and PREMs (Patient-Reported Experience Measures) for complex patients with diabetic foot, underscoring the need for their development. Even in the case of lower limb amputation—one of the most reliably tracked procedures in administrative data—its applicability for public health decisions remains limited [84]. A recent review of international knowledge exchange methods has proposed a step-wise approach to improve quality assessment in diabetes care. This approach should be adapted to a country’s resources—whether high-, middle-, or low-income—beginning with basic prevalence measurement and advancing to the evaluation of care in primary and tertiary facilities [82]. While manual audits in low-income countries yield valuable insights for care improvement, a careful balance is needed to protect clinician-patient time.

We must ensure that data-driven healthcare does not exacerbate existing disparities. If prediction models are trained on data from only a privileged subset of the population, their recommendations will be biased and less effective for minority or underserved groups. To build sustainable solutions, underserved regions must be supported, and their epidemiological and socioeconomic data must be included in the development processes of global AI health networks [69]. AI-driven innovations hold significant potential for addressing health disparities and promoting culturally sensitive, accessible care [85], a promise supported by emerging evidence of their transformative impact even in low-resource settings [86].

Faster progress is contingent upon strong systemic change. The emerging EHDS [26] represents an example of a powerful catalyst for providing internationally comparable health data. It establishes systemic support for a European common data ecosystem with a concomitant framework for data reuse, promoting a secure, holistic structure for international data access and sharing. While the EHDS is a tremendous step in the right direction—with numerous obligatory diabetes parameters defined in its priority categories (patient summaries, electronic prescriptions and dispensations, medical imaging studies and related reports, medical test results, and discharge reports)—it still lacks many specific data elements for diabetic foot care. A critical unmet need is the international standardization and consistent definition of these data elements for integration into a wider framework. International professional societies should play a crucial role by supplementing the current practice, in which such standards are largely created by different software vendors in an uncoordinated manner.

### 5.1. Support for Local Insights and FAIR Quality Measures

Local teams cannot rely solely on external studies and require deeper insights tailored to their needs and to specific populations. The SCORE2-Diabetes study [87] effectively demonstrated the importance of such population-specific adjustments for generating meaningful evidence. To empower local teams, the Observational Health Data Sciences and Informatics (OHDSI) initiative [18] provides a foundational analytics ecosystem built on the OMOP/CDM and standardized medical vocabularies. This framework transforms disparate, raw healthcare data—from electronic health records or claims—into a consistent, structured format using a universal language. Standardization ensures that the same analytical code can be run reliably across different databases, enabling reproducible, large-scale research. For the analytical process itself, OHDSI offers a comprehensive suite of open-source tools [88]. This platform represents a powerful element of the emerging common data ecosystem and enables researchers to perform everything from simple cohort characterization to complex population-level estimation studies without writing code, all within a standardized framework that ensures consistency and transparency throughout the research lifecycle.

Current creation of international quality measures is often predicated on a one-size-fits-all approach that assumes the applicability of high-income clinical standards across diverse settings. To be effective, quality measures must be adapted to a country’s specific context, health system capacity, and competing priorities [82]. To enhance the usability and transparency of various indicators, they must be supplemented with contextual metadata about the data and environment from which they draw and the related data processes. Future efforts should focus on developing methodologies to represent such rich contextual information alongside the indicator values themselves [89]. These methodologies should be guided by the FAIR principles to ensure the contextual data and metadata about quality measures are Findable, Accessible, Interoperable, and Reusable (FAIR) and that they include clear and coherent quality descriptions [90]. Applying FAIR principles to quality measures would enhance their machine readability and overcome the current fragmentation, where indicators about care are often represented in unstandardized forms across diverse and scattered sources [91].

### 5.2. Utilizing Real World Data

Real-world data (RWD) provide a valuable and rich data source beyond the confines of traditional epidemiological studies, clinical trials, and lab-based experiments, with lower cost in data collection compared to the latter [92]. Combining multiple RWD sources (e.g., linking EHR data with claims data or registry data) creates a more complete picture of a treatment’s real-world effectiveness, safety, and economic impact, which is essential for informed decision-making in healthcare [93]. RWD and real-world evidence (RWE) are poised to see increased application, given the abundance of data from various sources. Standard RCTs alone can not address the complex, patient-centric intersection of multiple diseases and comorbidities. This gap necessitates alternative methods for generating evidence. To develop better insights into care dynamics and outcomes, analytical initiatives are seeking to augment administrative and statistical data with clinical data from primary and specialized healthcare [94]. This will enable a more precise, patient-centric understanding of value and effectiveness. The major challenge again remains standardizing and validating such data to ensure its reliability for Health technology assessment. Achieving consensus on data quality frameworks, study design, and analysis standards will likely have a greater short-term impact on the adoption of RWE than the creation of new RWD sources.

## 6. Limitations

This narrative review synthesizes key concepts in modern digital health ecosystems. Given the interdisciplinary and rapidly evolving nature of topics like EHR usability, interoperability standards, and AI, a traditional systematic review methodology was not feasible. Instead, the review is based on a purposeful sampling of recent literature, guided by the authors’ expertise and judgments of thematic relevance. Consequently, its recommendations may be subjective.

The discussion on systemic change for interoperability is framed primarily through the emerging EHDS. This perspective may not fully address the specific contexts of low- and middle-income countries, as a fully encompassing global framework would need to integrate a wider array of regional approaches and socioeconomic circumstances [95,96]. Nonetheless, adopting internationally recognized standards, as exemplified by the EHDS, can provide a beneficial foundation for system development also in low- and middle-income countries contexts [97].

Although this methodology enabled a broad synthesis, it is not an exhaustive or bias-free inventory of the literature. Addressing the identified gaps requires targeted capacity building, improved literacy, and connecting disparate initiatives. Therefore, a critical next step is to foster broad stakeholder consensus to create a concrete action plan for improving the real usability of interoperable systems in diabetic foot care. This text is an initial attempt to stimulate the debate needed to build that consensus.

## 7. Conclusions

In conclusion, in the current situation, the improvement of healthcare through data for diabetic foot care is a multi-faceted journey. It requires a strong foundation of clean, integrated data; powerful analytical engines to find insights; and a relentless focus on deploying those insights to improve patient outcomes, operational efficiency, and the daily work of healthcare professionals. We must move beyond viewing EHR systems as mere repositories of patient data and instead envision them as intelligent partners in care delivery. With implementation steered towards systems fit for practice, local teams need strong systemic support and should be meaningfully included in proper governance. Such transformation will require concerted effort from multiple stakeholders: clinicians must articulate their needs more clearly, vendors must prioritize usability over checkbox features, and policymakers must create incentives for meaningful use rather than just documentation completeness.

This review has explored prominent possibilities for improving data management in diabetic foot care. However, the analysis is not comprehensive, given the rapidly evolving nature of the field and the lack of established consensus. The prize for getting this right is substantial—healthcare systems where technology amplifies rather than impedes clinical judgment, where administrative burdens recede rather than grow, and where patients and providers can focus on what matters most: delivering and receiving excellent care. In the context of diabetic foot care, where the paramount goals are tissue preservation and amputation prevention [98], collaborative data management must be recognized as a critical treatment modality itself. “Data is tissue”. It is the foundational element that enables the timely, coordinated, and evidence-based interventions necessary for success.

## Figures and Tables

**Table 1:** The Four Layers in the Knowledge Representation Framework [54]; CDS—clinical decision support.

Layer	Narrative(L1)	Semi- Structured(L2)	Structured(L3)	Executable(L4)
**Format**	Narrative text	Organized text	Coded and interpretable by computer	Coded and interpretable by CDS systems; variety of formats
**Shareability** **of Knowledge**	Broad	Broad	Broad	Very limited
**CDS Modality** **and Tool** **Independent**	Yes	Yes	Yes	No
**Site** **Independent**	Yes	Yes	Yes	No
**Author**	Guideline developer	Clinical domain expert	Knowledge engineer	CDS implementer
**Purpose**	Communication of policy; synthesis of evidence	Recommendations for implementation in CDS	Precise communication; validation	Implementation for a particular site

## Data Availability

Not applicable.

## Abbreviations

The following abbreviations are used in this manuscript:

AI	Artificial Intelligence
CDM	Common Data Model
CPG	Clinical Practice Guidelines
EEHRxF	European Electronic Health Record Exchange Format
EHDS	European Health Data Space
EHR	Electronic Health Record
EUBIROD	European Best Information Through Regional Outcomes in Diabetes
FAIR	Findable, Accessible, Interoperable, and Reusable
FHIR	Fast Healthcare Interoperability Resources
HCP	Healthcare Professionals
ICHOM	International Consortium for Health Outcomes Measurement
IDF	International Diabetes Federation
IT	Information Technology
IWGDF	International Working Group on the Diabetic Foot
LLM	Large Language Model
LOINC	Logical Observation Identifiers, Names, and Codes
OECD	Organization for Economic Co-operation and Development
OHDSI	Observational Health Data Sciences and Informatics
OMOP	Observational Medical Outcomes Partnership
ORPHA	Orphanet Rare Disease Ontology
OpenEHR	Open Electronic Health Record
RWD	Real World Data
RWE	Real World Evidence
SNOMED CT	Systematized Nomenclature of Medicine—Clinical Terms
WHO	World Health Organization
